# Evaluating Reproducibility and Transparency in Emergency Medicine Publications

**DOI:** 10.5811/westjem.2021.3.50078

**Published:** 2021-07-14

**Authors:** Bradley S. Johnson, Shelby Rauh, Daniel Tritz, Michael Schiesel, Matt Vassar

**Affiliations:** * Oklahoma State University, Center for Health Sciences, Tulsa, Oklahoma; † Oklahoma State University Medical Center, Department of Emergency Medicine, Tulsa, Oklahoma

## Abstract

**Introduction:**

We aimed to assess the reproducibility of empirical research by determining the availability of components required for replication of a study, including materials, raw data, analysis scripts, protocols, and preregistration.

**Methods:**

We used the National Library of Medicine catalog to identify MEDLINE-indexed emergency medicine (EM) journals. Thirty journals met the inclusion criteria. From January 1, 2014–December 31, 2018, 300 publications were randomly sampled using a PubMed search. Additionally, we included four high-impact general medicine journals, which added 106 publications. Two investigators were blinded for independent extraction. Extracted data included statements regarding the availability of materials, data, analysis scripts, protocols, and registration.

**Results:**

After the search, we found 25,473 articles, from which we randomly selected 300. Of the 300, only 287 articles met the inclusion criteria. Additionally, we added 106 publications from high-impact journals of which 77 met the inclusion criteria. Together, 364 publications were included, of which 212 articles contained empirical data to analyze. Of the eligible empirical articles, 2.49%, (95% confidence interval [CI], 0.33% to 4.64%] provided a material statement, 9.91% (95% CI, 5.88% to 13.93%) provided a data statement, 0 provided access to analysis scripts, 25.94% (95% CI, 20.04% to 31.84%) linked the protocol, and 39.15% (95% CI, 32.58% to 45.72%) were preregistered.

**Conclusion:**

Studies in EM lack indicators required for reproducibility. The majority of studies fail to report factors needed to reproduce research to ensure credibility. Thus, an intervention is required and can be achieved through the collaboration of researchers, peer reviewers, funding agencies, and journals.

## INTRODUCTION

Reproducible research is a hallmark of the scientific enterprise. The National Science Foundation defines reproducibility as “the ability of a researcher to duplicate the results of a prior study using the same materials and procedures” and considers reproducibility “a minimum necessary condition for a finding to be believable and informative.”^1^,^2^ Similarly, the National Institutes of Health (NIH) has implemented a rigor and reproducibility initiative for federally funded studies after NIH leadership called for “immediate and substantive action” to be taken to address the reproducibility crisis.^3^ Reproducibility occurs when independent investigators are able to validate a study’s findings using resources such as raw data, analysis scripts, study materials, and the protocol provided by the original investigators,^2^ and it is crucial to establishing credible and reliable research that governs clinical practice.

The current reproducibility problem in biomedical literature is cause for concern because up to 90% of preclinical research may not be reproducible.^4^ The reproducibility of emergency medicine (EM) studies is unclear and warrants further attention. A 2017 study found that only 4% of simulation-based education studies—which comprise one-quarter of all EM studies—provided the materials necessary to reproduce the intervention.^5^ Niven et al^6^ conducted a scoping review of reproducibility attempts for clinical studies in the critical care literature and reported that more than half of these attempts failed to demonstrate effects consistent with the original investigation. Thus, the limited available evidence calls into question the reproducibility of EM research.

The importance of reproducible findings is well illustrated by the controversy within the EM community over research on tissue plasminogen activator (tPA) for acute ischemic stroke. Some emergency physicians believe that of the 13 major randomized controlled trials conducted to evaluate the efficacy of tPA in stroke patients, only two provided evidence supporting its use. Among the 11 remaining studies, seven found no significance, three were terminated prematurely because of patient harm, and one provided evidence of increased mortality.^7^,^8^ However, the current clinical guidelines from the American College of Emergency Physicians recommend the use of tPA with moderate clinical certainty.^9^ Relying heavily on evidence from the two major trials with positive results, which have not been reproduced in the other 11 major trials, showcases the importance of reproducibility to generate stable results because standards of care may be affected.

Given the recent attention to the reproducibility crisis in science and the limited knowledge of study reproducibility in the EM literature, we undertook an investigation to explore the current climate of reproducible research practices within the EM research community. We applied indicators of reproducible research practices developed by Hardwicke et al^10^ to a broad, random sample of EM literature to evaluate whether investigators used reproducible research practices and provided necessary documentation for subsequent reproduction attempts. Ultimately, results from this investigation may serve as baseline data to determine whether reproducible practices improve over time.

## MATERIALS AND METHODS

This study was observational and used a cross-sectional design based upon the methodology of Hardwicke et al,^10^ with modifications. Our study is reported in accordance with guidelines for meta-epidemiological methodology research.^11^ To aid in reproducibility, we have uploaded pertinent materials for this study onto the Open Science Framework (https://osf.io/n4yh5/).

### Journal and Study Selection

We used the National Library of Medicine (NLM) catalog to search for all journals, using the subject terms tag “Emergency Medicine[ST].” This search was performed on May 29, 2019. The inclusion criteria required that journals were in English and MEDLINE-indexed. The final list of journals had the electronic International Standard Serial Number (ISSN) extracted (or linking ISSN if electronic was unavailable) to be used in a PubMed search. The PubMed search was performed on May 31, 2019. We limited our search to studies published from January 1, 2014–December 31, 2018. From the final list of studies and using the RANDBETWEEN function in Excel (Microsoft Corp., Redmond, WA), we assigned a random number to each and sorted them from lowest to highest value. The top 300 studies were chosen to be coded with additional studies available if necessary. Studies found from our search string are found: (https://osf.io/2964g/).

After peer review, we expanded the search strategy to include EM publications from high-impact factor general medicine journals. These four non-EM journals (*New England Journal of Medicine, Lancet, Journal of the American Medical Association, and British Medical Journal*) were based on Google Scholar Metrics and the H-5 index. PubMed was searched using these journals and a search string based on one from Brown et al^12^. We have included the exact search string here: (https://osf.io/rg8f5/). From this search, a total of 106 EM publications from the four non-EM journals were sampled.

### Data Extraction Training

Two investigators assigned to data extraction (BJ and SR) underwent a full day of training to ensure reliability. The training began with an in-person session that familiarized the two investigators with the standardized protocol, Google extraction from, and areas for which data may be located within two standardized practices publications. The authors were given three example articles from which to extract data independently. Following extraction, the investigators reconciled differences between data. This training session was recorded and listed online for reference (https://osf.io/tf7nw/). As a final training example, the investigators extracted data from the first 10 articles in their specialty list followed by a final consensus meeting. Data extraction on the remaining 290 articles was then conducted. A final consensus meeting was held by the pair to resolve disagreements in which the investigators were able to reference the original articles to settle disputes. A third author was available for adjudication, if necessary.

### Data Extraction

Data extraction on the remaining 290 articles was conducted in a duplicate and blinded fashion. A final consensus meeting was held by the pair to resolve disagreements. A third author (DT or MV) was available for adjudication but was not required. A pilot-tested Google Form was created based on the one provided by Hardwicke et al,^10^ with additions. This form prompted coders to identify whether a study had important information necessary to be reproducible, such as the availability of data, materials, protocols, and analysis scripts (https://osf.io/3nfa5/). The data extracted varied, based on the study design, with studies having no empirical data being excluded (eg, editorials, commentaries [without reanalysis], simulations, news, reviews, and poems). In our form, we included the five-year impact factor, if available, and the impact factor for the most recent year found. We also expanded the options of the study design to include cohort, case series, secondary analysis, chart review, and cross-sectional. Finally, we increased the funding options from public, private, or mixed to be more specific, such as university, hospital, public, private/industry, nonprofit, and mixed.

### Open Access Availability

Open access evaluation is a necessary aspect of our reproducibility analysis due to paywalls preventing others from accessing the components of reproducibility. We analyzed publications for accessibility through the openaccessbutton.org. Investigators used publication’s title or digital object identifier (DOI) to search the open access website. If openaccessbutton.org was not successful in providing access to the manuscript, the investigators searched for access through Google (https://www.google.com/) and PubMed (https://www.ncbi.nlm.nih.gov/pubmed/).

### Statistical Analysis

We report descriptive statistics for each category with 95% confidence intervals (CI), using Microsoft Excel.

## RESULTS

### Sample Characteristics

Our search of the NLM catalog identified 52 journals, with only 30 meeting our inclusion criteria. The ISSN for each of these journals was used in a PubMed search, yielding 90,380 publications. For this analysis, we included 25,473 publications from January 1, 2014–December 31, 2018. We randomly sampled 300 publications from this list. Additionally, we included a second search string that resulted in 106 publications from the *New England Journal of Medicine, Lancet, JAMA*, and *BMJ* to be added to our analysis.

**Figure 1:**
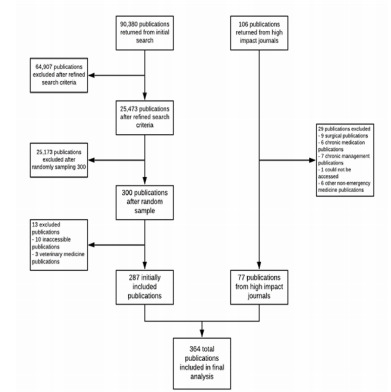
Flow diagram for included and excluded studies.

We assessed articles from EM journals with a broad range of most recently available impact factors (median 2.333, range 1.408 to 5.441). A total of 406 articles were assessed, with 364 eligible for inclusion. The 42 ineligible article had full texts that were inaccessible or were not related to EM (Figure 1). Other sample characteristics are presented in Table 1.

### Reproducibility and Related Characteristics

The number of studies that included each indicator of reproducibility and the significance of the indicator can be found in Supplementary Table 1. Among the 364 eligible articles, 122 (33.52%; 95% CI, 28.67% to 38.37%) were publicly available through the OpenAccess website, 127 (34.89%; 95% CI, 29.99% to 39.79%) were accessible through other means, and 115 (31.59%; 95% CI, 26.81% to 36.37%) were accessible only through a paywall. A variety of tools are used to describe how a research study is performed, including research protocols (may include the hypothesis, methods, and analysis plan) and research materials (may include equipment, questionnaire items, stimuli, computer programs, etc). Of the 364 eligible articles, 212 had study designs capable of including a protocol and data availability statement, providing analysis scripts, and being preregistered. Fifty-five of the 212 (25.94%; 95% CI, 20.04% to 31.84%) articles contained a statement about protocol availability. In addition, 191 of the 212 (90.09%; 95% CI, 86.07% to 94.11%) did not include a statement about data availability. Analysis scripts are needed for step-by-step documentation of how an analysis was performed. None of the 212 examined articles contained a reference to the analysis script. Preregistration is the process of documenting a time-stamped, read-only study protocol in a public database such as ClinicalTrials.gov prior to the start of the study. Eighty-three (39.15%; 95% CI, 32.58% to 45.72%) of the 212 examined articles were preregistered. Lastly, 201 of the 364 were study designs capable of containing a materials availability statement in which five of the (2.49%; 95% CI, 0.33% to 4.64%) articles reported a materials availability statement (Table 2). Supplementary Table 2 includes the percentage of publications that contain each reproducibility indicator from each journal. Additionally, we compared those findings to journal requirements found on each journal’s “guide for authors” webpage.

### Conflict of Interest and Funding Statements

Statements regarding conflicts of interest and funding sources are needed to assess the possibility of bias of the study’s authors. Of the 364 examined articles, 62 (17.03%; 95% CI, 13.17% to 20.89%) stated that there were one or more conflicts of interest, 170 (46.70%; 95% CI, 41.55% to 51.82%) stated that there was no conflict of interest, and 132 (36.26%; 95% CI, 31.32% to 41.20%) did not include a conflict-of-interest statement (Table 2). Of the 364 included articles, 47 (12.91%; 95% CI, 9.47% to 16.36%) stated that there was no funding received and 190 (52.20%; 95% CI, 47.07% to 57.33%) did not include a funding statement. The remaining articles included detailed statements about their funding sources, detailed in Table 1.

### Replication and Evidence Synthesis

Replication studies help to ensure the validity and reliability of previous scientific claims made by research studies by replicating the methodology used in novel studies. None of the examined publications self-reported that it was a replication study. On a large scale, evidence that is gathered across numerous studies related to a single topic can be aggregated and synthesized through meta-analyses and systematic reviews. Of the 241 examined articles, 153 (63.49%; 95% CI, 57.41% to 69.56%) of the examined articles were not included in a meta-analysis or systematic review (Table 2).

## DISCUSSION

Our findings indicate that EM studies lack the components needed for reproducibility. Overall, the studies in our sample were deficient in most of the reproducibility and transparency indicators, such as data availability, material availability, and protocol availability. In addition, we found no replication attempts. Thus, the current climate of EM research is not indicative of such practices.

Our analysis indicated that only 3% of publications provided a materials availability statement and only 1 in 4 publications made protocols readily accessible. Similar trends have been seen in other areas of medicine.^10^,^13^ A lack of access to full protocols and materials used during a study, may hinder reproducibility of experiments. Furthermore, data availability statements were lacking among EM studies, with only 13 providing statements that offered at least partial data. Ultimately, nearly all EM studies in our sample gave no method for retrieving raw data. These findings are consistent with studies conducted in other disciplines.^13^,^14^ This trend is disappointing given the value of data sharing to study integrity.

Data availability allows readers to gain greater insight into a study’s methodology and may reveal inconsistencies between the raw data and the study conclusions. However, data sharing is a complex, multifactorial issue. Longo and Drazen,^15^ in a *New England Journal of Medicine* editorial, described the potential dangers of data sharing that may arise from “research parasites”— scientists who use others’ data for personal gain, without contributing to the methodology or execution of the study. These authors also argued that secondary investigators are not likely to understand the choices made when defining the parameters regarding the data (ie, differences in patient populations and special modifications to the protocol).^15^ Data sharing is also complicated and time consuming. A 2018 survey of 7700 scientists found that they had difficulty with data organization, data repository selection, and handling copyright issues.^16^ Also, some scientists indicated that the additional time needed to share their data was a challenge.^16^

We encourage researchers to gain familiarity with both the FAIR guiding principles for scientific data management and open science principles for data sharing. FAIR principles ensure the findability, accessibility, interoperability, and reusability of study data, placing an emphasis on data being provided to the right people, in the right way, and at the right time. In contrast, open data refers to unrestricted use of study data free of copyrights, patents, or licenses. An understanding of these principles will allow researchers to make their own informed decisions about sharing data and under what conditions sharing should occur. While the lack of data sharing is due to a whole host of reasons in general, a small portion of the studies that fail to provide raw data may be associated with unethical behavior.

The current research culture of “publish or perish”^17^ may entice credible entities to falsify data to compete for grant funding. For example, researchers at Duke University were discovered to be fabricating data in their grant application, resulting in $112 million in penalties in 2019.^18^ The court case revealed that none of the researchers’ data were reproducible from 2006–2013.^18^ A laboratory research analyst in the pulmonary asthma critical care division of the Duke University health system explained that members of the laboratory had trusted the results without verifying their raw data for over a decade.^19^ Overall, the cause for the small number of data availability statements in EM is complex but needs to be addressed.

**Figure 2:**
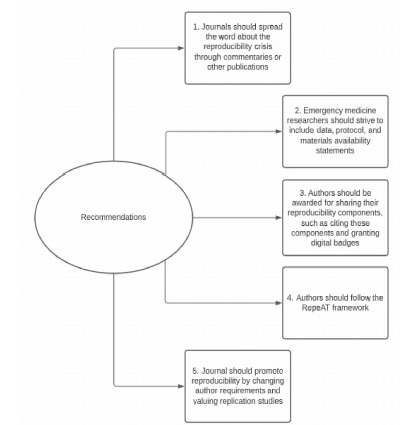
Recommendations for Promoting Reproducibility

Extensive evidence shows that a reproducibility problem exists within various fields of science and medicine.^4^–^6^ Our findings validate this problem within EM. However, this problem provides leaders within EM the opportunity to be pioneers for change within medical research. Most of the reproducibility problems stem from either motivation to remain competitive for research funding or the difficult, time-consuming nature of including all factors needed for reproducing a study. Additionally, it is important to include the journal’s role in the discussion of reproducibility as they ultimately dictate what authors share through journal requirements, word limits, and other restrictions. In fact, one study found that nearly two-thirds of the 799 scientists surveyed listed journal requirements as a motivator for data sharing.^20^ If authors are not expected to include reproducibility indicators, they would likely not include these indicators due to limited word count. This may result in the undesired removal of study details. The complexity of this reproducibility issue may explain why many experts have recommended solutions directed at the problem, but none have been completely successful. We propose a series of recommendations to help leaders in EM solve the reproducibility problem. This is outlined in Figure 2.

Our first recommendation is to spread the findings of our study and encourage journals to accept commentaries regarding the reproducibility crisis. Second, we recommend all EM studies consider including a statement of availability for data, protocol, and materials. Including availability statements is a tangible way for authors to improve the reproducibility of their studies. Evidence shows that when journals require authors to include a data availability statement, the authors share their data more often.^14^

Third, we recommend leaders in EM research to motivate authors to share their reproducibility components through reproducibility component citation and awarding digital badges. Allowing authors’ data, materials, and protocols to be cited in other studies provides an opportunity for authors to be rewarded for their efforts to enhance reproducibility because including raw data has been shown to increase the number of citations a study receives.^21^ Digital badges can be awarded to authors as a stamp of approval for reproducible science. A 2016 study revealed that digital badges significantly increased the sharing of raw data.^22^

Fourth, we encourage authors to use the RepeAT framework to help ensure that their study is reproducible. RepeAT is an experimental framework designed by experts in reproducibility to increase the reproducibility of a study through use of a list of 119 variables that can act as a checklist for authors. Taken from the RepeAT framework, Appendix 1 is a full list of the framework’s variables along with its relations to transparency or accessibility.^23^ RepeAT can be an additional resource for authors to ensure they include everything that is needed for reproducible research. Finally, we encourage EM journals to promote reproducibility by changing journal policies and author instructions to include reproducibility indicators. These journals can also promote reproducibility by valuing replication studies as they do novel findings because both have the potential to change the standard of care. There are numerous examples of studies having altered clinical practice and later found to be harmful.^24^ Reproducibility encourages the replication and validation of a study’s findings prior to influencing change in clinical practice guidelines. These recommendations are backed by expert opinion and evidence, which we hope will help leaders in EM to act to solve the reproducibility problem in their field.

## LIMITATIONS

Our study has many strengths, including double-blinded data extraction, to ensure any bias was limited. The dual data extraction is considered a gold standard practice in meta-research.^25^ However, we acknowledge limitations within our study. For example, our analysis only included studies taken from a certain time period, possibly limiting the ability to generalize our results to studies outside that time period. Next, we did not attempt to retrieve any of the components of reproducibility from the authors directly. Authors may have made these components available or given us an adequate explanation for exclusion. However, for the sake of feasibility, we believe our methods are adequate for describing the trend of irreproducibility within EM.

## CONCLUSION

Reproducibility in EM research is lacking in many indicators studied. To ensure that reliable research drives the standard of care we outline a plan that includes informing experts in EM about the reproducibility problem, requiring authors to include an availability statement, helping authors to include everything needed for reproducible research, providing incentives for authors, and giving a reason for journals to value reproducibility more. Emergency medicine journals and researchers must promote reproducibility to maintain and assure the credibility of research.

**Population Health Research Capsule**

What do we already know about this issue?

*Most biomedical research cannot be reproduced due to lack of key information (ie, reproducibility indicators) in the published research*.

What was the research question?

*Does this relationship of lack of reproducibility indicator sharing and irreproducible research hold true in emergency medicine (EM)?*

What was the major finding of the study?

*Nearly all of EM research is lacking indicators of reproducibility which makes assessing the reliability of EM research and its findings difficult*.

How does this improve population health?

*By addressing irreproducibility, others can confirm EM research findings through reproducing a study. This is important as EM research dictates the standard of care in EM*.

## Supplementary Information







## Figures and Tables

**Table 1:** Characteristics of included publications.

Characteristics	Variables	

	N (%)	95% CI
Funding (N=364)		
University	5 (1.37)	0.18–2.57%
Hospital	7 (1.92)	0.51–3.33%
Public	46 (12.64)	9.22–16.05%
Private/Industry	23 (6.32)	3.82–8.82%
Non-Profit	3 (0.82)	0–1.75%
Mixed	43 (11.81)	8.50–15.13%
No Statement Listed	190 (52.20)	47.07–57.33%
No Funding Received	47 (12.91)	9.47–16.36%
Type of study (N=364)		
No Empirical Data	112 (30.77)	26.03–35.51%
Meta-Analysis	10 (2.74)	1.07–4.43%
Commentary	1 (0.27)	0–0.81%
Clinical Trial	84 (23.08)	18.75–27.41%
Case Study	38 (10.44)	7.30–13.58%
Case Series	2 (0.55)	0–0.75%
Cohort	75 (20.60)	16.45–24.76%
Case Control	1 (0.27)	0–0.81%
Survey	20 (5.49)	3.15–7.84%
Laboratory	1 (0.27)	0–0.81%
Other	20 (5.49)	3.15–7.84%
5-Year impact factor (N=241)		
Median	2.333	-
1st Quartile	1.408	-
3rd Quartile	5.441	-
Interquartile Range	1.408 – 5.441	-

*CI*, confidence interval.

**Table 2:** Additional characteristics of reproducibility in emergency medicine studies.

Characteristics	Variables	

	N (%)	95% CI
Open access (N=364)		
Yes - found via Open Access Button.com	122 (33.52)	28.67–38.37%
Yes - found article via other means	127 (34.89)	29.99–39.79%
Could not access through paywall	115 (31.59)	26.82–36.37%
Protocol availability (N=212)		
Full Protocol	55 (25.94)	20.04–31.84%
No Protocol	157 (74.06)	68.16–79.96%
Data availability (N=212)		
Statement, some data are available	13 (6.13)	2.90–9.36%
Statement, data are not available	8 (3.77)	1.21–6.34%
No data availability statement	191 (90.09)	86.07–94.12%
Analysis Script Availability (N=212)		
Statement, analysis scripts are not available	0	-
No analysis script availability statement	212	-
Pre-registration (N=212)		
Statement, says was pre-registered	83 (39.15)	32.58–45.72%
Statement, says was not pre-registered	1 (0.47)	0–1.39%
No, there is no pre-registration statement	128 (60.38)	53.79–66.96%
Material availability (N=201)		
Statement, some materials are available	5 (2.49)	0.33–4.64%
Statement, materials are not available	0	-
No materials availability statement	196 (97.51)	95.36–99.67%
Conflict of interest statement (N=364)		
Statement, one or more conflicts of interest	62 (17.03)	13.17–20.89%
Statement. no conflict of interest	170 (46.70)	41.58–51.83%
No conflict-of-interest statement	132 (36.26)	31.32–41.20%
Replication studies (N=212)		
Novel study	211 (99.53)	-
Replication	1 (0.47)	-
Cited in a systematic review/meta-analysis (a) (N=212)		
No citations	153 (72.17)	66.14–78.20%
A single citation	22 (10.38)	6.27–14.48%
One to five citations	8 (3.77)	1.21–6.34%
Greater than five citations	26 (12.26)	7.85–16.68%
a - No studies were explicitly excluded from the systematic reviews or meta-analyses that cited the original article.		
Most recent impact factor year (N=364)		
2014	0	-
2015	0	-
2016	0	-
2017	242	-
2018	85	-
Not found	37	-

## References

[1] Cook FL (2016). Dear Colleague Letter: Robust and Reliable Research in the Social, Behavioral, and Economic Sciences.

[2] Goodman SN, Fanelli D, Ioannidis JPA (2016). What does research reproducibility mean?. Sci Transl Med.

[3] Collins FS, Tabak LA (2014). Policy: NIH plans to enhance reproducibility. Nature.

[4] Begley CG, Ioannidis JP (2015). Reproducibility in science: improving the standard for basic and preclinical research. Circ Res.

[5] Chauvin A, Truchot J, Bafeta A (2018). Randomized controlled trials of simulation-based interventions in emergency medicine: a methodological review. Intern Emerg Med.

[6] Niven DJ, McCormick TJ, Straus SE (2018). Reproducibility of clinical research in critical care: a scoping review. BMC Med.

[7] Morgenstern J (2017). Thrombolytics for stroke: The evidence.

[8] Rezaie S (2019). Extending the tPA window to 4.5 – 9 hours in acute ischemic stroke (AIS)?.

[9] Carneiro T, Dashkoff J, Leung LY (2020). Intravenous tPA for Acute Ischemic Stroke in Patients with COVID-19.

[10] Hardwicke TE, Wallach JD, Kidwell MC (2020). An empirical assessment of transparency and reproducibility-related research practices in the social sciences (2014–2017). R Soc Open Sci.

[11] Murad MH, Wang Z (2017). Guidelines for reporting meta-epidemiological methodology research. Evid Based Med.

[12] Brown J, Lane A, Cooper C (2019). The results of randomized controlled trials in emergency medicine are frequently fragile. Ann Emerg Med.

[13] Iqbal SA, Wallach JD, Khoury MJ (2016). Reproducible research practices and transparency across the biomedical literature. PLoS Biol.

[14] Alsheikh-Ali AA, Qureshi W, Al-Mallah MH (2011). Public availability of published research data in high-impact journals. PLoS One.

[15] Longo DL, Drazen JM (2016). Data sharing. N Engl J Med.

[16] Gimpel J (2018). Researchers’ challenges in sharing data cross geographic borders and disciplines.

[17] Fogarty PP (2018). Publish, perish, or put patients first?. Int J Gynaecol Obstet.

[18] Dyer O (2019). Duke University pays $112m to settle claims that it knowingly used falsified data to obtain research grants. BMJ.

[19] United States District Court for the Western District of Virginia Danville Division (2017). The United States of America ex rel., et al. v. Duke University, et al., No. 4:2013cv00017 - Document 107 (W.D. Va. 2017).

[20] Enke N, Thessen A, Bach K (2012). The user’s view on biodiversity data sharing — Investigating facts of acceptance and requirements to realize a sustainable use of research data —. Ecological Informatics.

[21] Piwowar HA, Day RS, Fridsma DB (2007). Sharing detailed research data is associated with increased citation rate. PLoS One.

[22] Kidwell MC, Lazarević LB, Baranski E (2016). Badges to acknowledge open practices: a simple, low-cost, effective method for increasing transparency. PLoS Biol.

[23] McIntosh LD, Juehne A, Vitale CRH (2017). Repeat: a framework to assess empirical reproducibility in biomedical research. BMC Med Res Methodol.

[24] Prasad V, Cifu A (2011). Medical reversal: why we must raise the bar before adopting new technologies. Yale J Biol Med.

[25] Green S, Higgins J, Alderson P Cochrane Handbook for Systematic Reviews of Interventions.

