# Supplementary material for: Evaluating Reproducibility and Transparency in Emergency Medicine Publications
Source: West J Emerg Med. 2021 Jul 14;22(4):963–71. doi: 10.5811/westjem.2021.3.50078 (PMC8328179; doi:10.5811/westjem.2021.3.50078)
Supplement: Supplementary file 3 [file wjem-22-963-s003.docx]

| **Supplementary Table 2: The Percentage of Studies that Reported the Following Reproducibility Indicators in Comparison to Each Journal's Requirements** | | | | | | | |
| --- | --- | --- | --- | --- | --- | --- | --- |
| **Journal** | **2017/2018 Impact Factor** | **Materials Availability Statement** | **Data Availability Statement** | **Protocol Availability** | **Analysis Scripts** | **Preregistered Trials** | **Journal Requirements** |
| New England Journal of Medicine | 70.331* | 0% (0/27) | 0% (0/27) | 88.89% (24/27) | 0% (0/27) | 88.89% (24/27) | Requires a data sharing statement and trial preregistration; Asks authors to submit protocols |
| Lancet | 54.664* | 0% (0/15) | 6.67% (1/15) | 33.33% (5/15) | 0% (0/15) | 93.33% (14/15) | Requires trial preregistration, protocols, and as of July 1, 2018, a data sharing statement |
| JAMA | 46.312* | 0% (0/27) | 3.70% (1/27) | 70.40% (19/27) | 0% (0/27) | 96.30% (26/27) | Requires a data sharing statement, a protocol, and trial preregistration; Suggests submitting statistical code in supplement material |
| BMJ | 24.546* | 0% (0/8) | 62.50% (5/8) | 37.50% (3/8) | 0% (0/8) | 100% (8/8) | Requires a data sharing statement, and trial preregistration; Asks for additional materials for replication purposes and a protocol |
| Annals of Emergency Medicine | 5.441 | 0% (0/10) | 7.69% (1/13) | 0% (0/13) | 0% (0/13) | 15.38% (2/13) | Requries trial preregistration and protocol submission |
| Resuscitation | 5.244 | 0% (0/14) | 37.50% (6/16) | 6.25% (1/16) | 0% (0/16) | 12.50% (2/16) | Requires trial preregistration; Encourages the sharing of data, protocols, materials, and analytic code/scripts for reproducibility |
| Academic Emergency Medicine | 2.947 | 11.11% (1/9) | 10% (1/10) | 0% (0/10) | 0% (0/10) | 10% (1/10) | Requires trial preregistration |
| Scandinavian Journal of Trauma, Resuscitation and Emergency Medicine | 2.612 | 0% (0/3) | 0% (0/3) | 66.66% (2/3) | 0% (0/3) | 66.66% (2/3) | Requires data availability statements, material availability statements, and trial preregistration; Encourages protocol submission |
| Internal and Emergency Medicine | 2.333 | 0% (0/3) | 0% (0/3) | 0% (0/3) | 0% (0/3) | 0% (0/3) | Encourages a data availability statement |
| Prehospital Emergency Care | 2.316 | 0% (0/4) | 0% (0/4) | 0% (0/4) | 0% (0/4) | 0% (0/4) | Requires trial preregistration; Asks for a data availability statement |
| Emergency Medicine Journal - BMJ | 2.007 | 0% (0/3) | 20% (1/5) | 0% (0/5) | 0% (0/5) | 0% (0/5) | Requires a data availability statement and trial preregistration |
| European Journal of Emergency Medicine | 1.729 | 0% (0/5) | 0% (0/5) | 0% (0/5) | 0% (0/5) | 0% (0/5) | Requires a data availability statement and trial preregistration |
| European Journal of Trauma and Emergency Surgery | 1.704 | 0% (0/5) | 0% (0/5) | 0% (0/5) | 0% (0/5) | 0% (0/5) | None |
| Canadian Journal of Emergency Medicine | 1.481 | 0% (0/2) | 50% (1/2) | 0% (0/2) | 0% (0/2) | 0% (0/2) | Requires trial preregistration |
| The American Journal of Emergency Medicine | 1.408 | 0% (0/19) | 10% (2/20) | 5% (1/20) | 0% (0/20) | 15% (3/20) | Encourages a data availability statement and a protocol |
| Emergency Medicine Australasia | 1.353 | 0% (0/3) | 0% (0/3) | 0% (0/3) | 0% (0/3) | 0% (0/3) | Asks for a data availability statement |
| The Journal of Emergency Medicine | 1.207 | 10% (1/10) | 0% (0/10) | 0% (0/10) | 0% (0/10) | 0% (0/10) | Encourages data availability statement; Encourages the sharing of data, protocols, materials, and analytic code/scripts for reproducibility |
| Wilderness & Environmental Medicine | 1.161 | 0% (0/2) | 0% (0/2) | 0% (0/2) | 0% (0/2) | 0% (0/2) | Requires trial preregistration |
| International Emergency Nursing | 1.093 | 0% (0/4) | 0% (0/4) | 0% (0/4) | 0% (0/4) | 0% (0/4) | Encourages a data availability statement; Encourages the sharing of data, protocols, materials, and analytic code/scripts for reproducibility |
| Pediatric Emergency Care | 1.066 | 9.09% (1/11) | 0% (0/11) | 0% (0/11) | 0% (0/11) | 0% (0/11) | None |
| Prehospital and Disaster Medicine | 1.01 | 25% (1/4) | 25% (1/4) | 0% (0/4) | 0% (0/4) | 0% (0/4) | Requires a study protocol |
| Journal of Emergency Nursing | 0.662 | 0% (0/1) | 0% (0/1) | 0% (0/1) | 0% (0/1) | 0% (0/1) | Encourages a data availability statement; Encourages the sharing of data, protocols, materials, and analytic code/scripts for reproducibility |
| Air Medical Journal | - | 0% (0/2) | 0% (0/2) | 0% (0/2) | 0% (0/2) | 0% (0/2) | Encourages a data availability statement; Encourages the sharing of data, protocols, materials, and analytic code/scripts for reproducibility |
| BMC Emergency Medicine | - | 0% (0/1) | 0% (0/1) | 0% (0/1) | 0% (0/1) | 0% (0/1) | Requires a data availability statement, material availability statement, and trial preregistration; |
| Emergency Radiology | - | 0% (0/2) | 0% (0/2) | 0% (0/2) | 0% (0/2) | 0% (0/2) | Encourages the sharing of data |
| Western Journal of Emergency Medicine | - | 0% (0/7) | 0% (0/7) | 0% (0/7) | 0% (0/7) | 0% (0/7) | None |
| * 2018 Impact Factor | | | | | | | |
| Differences in denominators between each reproducible indicator are due to the different number of expected indicators for each study type (ie. Normally, systematic reviews and meta-analyses are not expected to have materials) | | | | | | | |
| Journals, such as Emergency Medicine Clinics of North America, Advanced Emergency Nursing Journal, Emergency Medicine Practice, Emergency Nurse, and Journal of Emergency Management, were not included in this table due to lacking publication types that are reproducible in our sample (ie. editorials, case studies, etc.) | | | | | | | |
