# Supplementary material for: Evaluating Reproducibility and Transparency in Emergency Medicine Publications
Source: West J Emerg Med. 2021 Jul 14;22(4):963–71. doi: 10.5811/westjem.2021.3.50078 (PMC8328179; doi:10.5811/westjem.2021.3.50078)
Supplement: Supplementary file 3 [file wjem-22-963-s002.docx]

Supplemental Table 1: Additional Characteristics of Reproducibility in Emergency Medicine Studies

Characteristics		Variables N (%)	Variables 95% CI
Conflict of Interest Statement N=364	Statement, one or more conflicts of interest	62 (17.03)	13.17-20.89%
	Statement. no conflict of interest	170 (46.70)	41.58-51.83%
	No conflict of interest statement	132 (36.26)	31.32-41.20%
Data Availability (N=212)	Statement, some data are available	13 (6.13)	2.90-9.36%
	Statement, data are not available	8 (3.77)	1.21-6.34%
	No data availability statement	191 (90.09)	86.07-94.12%
Material Availability (N=201)	Statement, some materials are available	5 (2.49)	0.33-4.64%
	Statement, materials are not available	0	-
	No materials availability statement	196 (97.51)	95.36-99.67%
Protocol Availability (N=212)	Full Protocol	55 (25.94)	20.04-31.84%
	No Protocol	157 (74.06)	68.16-79.96%
Analysis Scripts N=212	Statement, some analysis scripts area vailable	0	-
	Statement, analysis scripts are not available	0	-
	No analysis script availability statement	212	-
Replication Studies N=212	Novel study	211 (99.53)	-
	Replication	1 (0.47)	-
Open Access N=364	Yes found via Open Access Button	122 (33.52)	28.67-38.37%
	Yes found article via other means	127 (34.89)	29.99-39.79%
	Could not access through paywall	115 (31.59)	26.82-36.37%
Most Recent Impact Factor Year N=364	2014	0	-
	2015	0	-
	2016	0	-
	2017	242	-
	2018	85	-
	Not Found	37	-

Characteristics		Variables N (%)	Variables 95% CI
Cited in a Systematic Review/ Meta-Analysis (a) (N=212)	No Citations	153 (72.17)	66.14-78.20%
	A Single Citation	22 (10.38)	6.27-14.48%
	One to Five Citations	8 (3.77)	1.21-6.34%
	Greater Than Five Citations	26 (12.26)	7.85-16.68%
a - No studies were explicitly excluded from the systematic reviews or meta-analyses that cited the original article.			
Pre-Registration (N=212)	Statement, says was pre- registered	83 (39.15)	32.58-45.72%
	Statement, says was not pre-registered	1 (0.47)	0-1.39%
	No, there is no pre-registration statement	128 (60.38)	53.79-66.96%
Test Subjects N=364	Animals	3 (0.82)	-
	Humans	237 (65.11)	-
	Both	0	-
	Neither	124 (34.07)	-
Country of Journal Publication N=364	US	267(73.35)	-
	UK	27 (7.42)	-
	Germany	13 (3.57)	-
	Canada	1 (0.27)	-
	Italy	15 (4.12)	-
	Unclear	12 (3.30)	-
	Other (a)	29 (7.97)	-
Country of Corresponding Author N=364	US	164 (45.05)	-
	China	9 (3.10)	-
	UK	19 (5.22)	-
	Germany	7 (1.92)	-
	Japan	9 (2.47)	-
	France	10 (2.75)	-
	Canada	27 (7.42)	-
	Italy	14 (3.85)	-
	India	1 (0.27)	-
	Spain	5 (1.37)	-
	Unclear	17 (4.67)	-
	Other	82 (22.53)	-

Characteristics		Variables N
Material Availability	Personal or institutional	0
	Supplementary information hosted by the journal	5
	Online third party	0
	Upon Request	0
	Yes, material was accessible	3
	No, material was not accessible	2
Data Availability	Personal or institutional	0
	Supplementary journal information	13
	Online third party	0
	Upon Request	6
	Other (b)	0
	Yes, data could be accessed and downloaded	10
	No, data count not be accessed and downloaded	9
	Yes, data files were clearly documented	9
	No, data files were not clearly documented	1
	Yes, data files contain all raw data	2
	No, data files do not contain all raw data	2
	Unclear if all raw data was available	6
Pre-Registration	Pre-registered on ClinicalTrials.gov	67
	Other (c)	16
	Yes, pre-registration was accessible	80
	No, pre-registration was not accessible	3
	Hypothesis was pre-registered	19
	Methods were pre-registered	13
	Analysis plan was pre-registered	4
Protocol	Hypotheses was included in the protocol	44
	Methods were included in the protocol	42
	Analysis plan was included in the protocol	44

Abbreviations: CI, Confidence Interval.
